# O-27 - DEVELOPING ACCESSIBLE, PLAIN LANGUAGE HEALTH PROTECTION COMMUNICATIONS: A MULTIDISCIPLINARY APPROACH

**DOI:** 10.1093/pubmed/fdag046.024

**Published:** 2026-07-09

**Authors:** Mr Rory Dinwoodie, Ms Rosie Allison, Ms Hayley Lightfoot, Ms Katherine Clark, Ms Emma Booth, Ms Alicia Thornton, Ms Lucy Wainwright, Ms Donna Gipson, Ms Amy Jackson

**Affiliations:** UK Health Security Agency; UK Health Security Agency; UK Health Security Agency; UK Health Security Agency; UK Health Security Agency; UK Health Security Agency; UK Health Security Agency; UK Health Security Agency; UK Health Security Agency

## Abstract

**Background:**

Limited literacy, health literacy, and digital literacy affect a substantial proportion of the UK population. Approximately half of adults have numeracy skills at or below primary school level; 7.1 million read at or below the level of an average nine-year-old; and up to one million people have limited English proficiency. However, health protection information is commonly written at a reading age of around 16 years and relies on medical jargon and complex sentence structures. This creates and exacerbates health inequalities, as populations most at risk are often least able to access or understand health information intended to keep them safe.

**Objectives:**

- To improve the accessibility, readability, and usability of health protection communications.
- To incorporate insights from people with lived experience alongside content designers and subject matter experts, while maintaining clinical accuracy.
- To develop a standard operating procedure (SOP) for accessible communication across infectious diseases and other health hazards.

**Methods:**

Content designers, health equity specialists, behavioural scientists, and regional health protection teams developed a standardised plain language template for health protection factsheets and warn-and-inform letters. In March 2025, two focus groups with people with lived experience and specific communication needs tested materials for tuberculosis and hepatitis A. Additional peer-led research with EP:IC informed resource development for STEC, typhoid and paratyphoid, and group A streptococcal infections.

**Results:**

Participants reported that highly formal or clinically complex communications increased anxiety and confusion. Plain language materials with clear structure, supportive tone, and accessible design features (e.g. headings, bullet points, colour use) were easier to understand, and increased confidence in following recommended public health actions.

**Conclusions:**

This work informed the development of plain language health protection resources and a supporting SOP, providing a consistent, evidence-based approach to accessible communication and supporting efforts to reduce health inequalities.

